# The Antimicrobial and Anti-Inflammatory Effects of Silver Nanoparticles Synthesised from *Cotyledon orbiculata* Aqueous Extract

**DOI:** 10.3390/nano11051343

**Published:** 2021-05-20

**Authors:** Caroline Tyavambiza, Abdulrahman Mohammed Elbagory, Abram Madimabe Madiehe, Mervin Meyer, Samantha Meyer

**Affiliations:** 1 Department of Biomedical Sciences, Cape Peninsula University of Technology, P.O. Box 1906, Bellville, Cape Town 7535, South Africa; carolinetyavambiza@gmail.com; 2 Chemistry Department, Cape Peninsula University of Technology, P.O Box 1906, Bellville, Cape Town 7535, South Africa; elbagorya@cput.ac.za; 3 DSI/Mintek Nanotechnology Innovation Centre, Department of Biotechnology, University of the Western Cape, Private Bag X17, Bellville, Cape Town 7535, South Africa; amadiehe@uwc.ac.za (A.M.M.); memeyer@uwc.ac.za (M.M.); 4 Nanobiotechnology Research Group, Department of Biotechnology, Faculty of Natural Sciences, University of the Western Cape, Private Bag X17, Bellville, Cape Town 7535, South Africa

**Keywords:** green nanotechnology, *Cotyledon orbiculata*, silver nanoparticles, nanoparticles characterisation, antimicrobial activity, immunomodulation, cytokines, anti-inflammation

## Abstract

*Cotyledon orbiculata*, commonly known as pig’s ear, is an important medicinal plant of South Africa. It is used in traditional medicine to treat many ailments, including skin eruptions, abscesses, inflammation, boils and acne. Many plants have been used to synthesize metallic nanoparticles, particularly silver nanoparticles (AgNPs). However, the synthesis of AgNPs from *C. orbiculata* has never been reported before. The aim of this study was to synthesize AgNPs using *C. orbiculata* and evaluate their antimicrobial and immunomodulatory properties. AgNPs were synthesized and characterized using Ultraviolet-Visible Spectroscopy (UV-Vis), Dynamic Light Scattering (DLS) and High-Resolution Transmission Electron Microscopy (HR-TEM). The antimicrobial activities of the nanoparticles against skin pathogens (*Staphylococcus aureus*, *Staphylococcus epidermidis*, Methicillin Resistance *Staphylococcus aureus*, *Pseudomonas aeruginosa* and *Candida albicans*) as well as their effects on cytokine production in macrophages (differentiated from THP-1 cells) were evaluated. The AgNPs from *C. orbiculata* exhibited antimicrobial activity, with the highest activity observed against *P. aeruginosa* (5 µg/mL). The AgNPs also showed anti-inflammatory activity by inhibiting the secretion of pro-inflammatory cytokines (TNF-alpha, IL-6 and IL-1 beta) in lipopolysaccharide-treated macrophages. This concludes that the AgNPs produced from *C. orbiculata* possess antimicrobial and anti-inflammation properties.

## 1. Introduction

*Cotyledon orbiculata* is a succulent plant indigenous to Southern Africa and is commonly found in the Western Cape province. It is a small shrub, commonly known as pig’s ear, that has thick green fleshy leaves with a red line around the edges of the leaves [1]. It is used in folk medicine to treat skin rashes, abscesses, inflammation, boils and acne [2,3]. The fleshy leaves are applied to corns and warts to soften them, and the leaf juice is used to treat earache, toothache and epilepsy [4]. Studies have shown that *C. orbiculata* have many biological activities, which include antibacterial, antifungal, anticonvulsant, antinociceptive, anti-inflammatory, antihelmintic and antioxidant activities [1,5,6,7]. Aremu and colleagues (2010) assessed the antimicrobial properties of several extracts (water, ethanol, dichloromethane and petroleum ether) of the *C. orbiculata* plant against *Staphylococcus aureus*, *Klebsiella pneumoniae*, *Escherichia coli*, *Bacillus subtilis* and *Candida albicans*. All the extracts showed antimicrobial activity, with Minimum Inhibitory Concentration (MIC) values ranging from 1.6 to 12.5 mg/mL [2]. Another study by Amabeoku and Kabatende (2012) showed that the administration of a methanol extract of the *C. orbiculata* leaf significantly attenuated carrageenan-induced oedema in the paws of rats, and thus demonstrated the anti-inflammatory properties of the plant extract [8].

Green nanotechnology is a division of nanotechnology which involves the synthesis of biogenic nanomaterials using eco-friendly methods that are safe and cost effective [9]. Microorganisms (bacteria, fungi and yeast) and plants have been used in the biosynthesis of biogenic nanoparticles [10]. The advantages of synthesizing nanoparticles by biological means include lower toxicity and easy scalability [11]. Plants have been readily used in the synthesis of metallic nanoparticles, as they are easily accessible and can promote rapid nanoparticle synthesis [12]. Metallic nanoparticles have been synthesized using plants such as *Galenia African*; *Hypoxis hemerocallidea* [10]; *Terminalia mantaly* [11]; *Capparis spinosa* [12]; *Datura stramonium* [13]; *Ocimum tenuiflorum*; *Citrus sinensis*; *Solanum tricobatum*; *Centella asiatica*; *Syzygium cumini* [14]; *Musa paradisiacal* [15]; *Aspalathus hispida*; *Aspalathus linearis*; *Asparagus rubicundus*; *Cynanchum africanum*; *Dicerothamnus rhinocertis*; *Eriocephalus africanus*; *Hermannia alnifolia*; *Indigofera brachystachya*; *Lobostemon glaber*; *Metalasia muricate*; *Nidorella foetida*; *Otholobium bracteolatum*; *Podocarpus falcatus*; *Podocarpus latifolius*; *Salvia africana-lutea*; *Senecio pubigerus*; *Searsia dissecta*; *Camellia sinensis*; and *Camellia sinensis* [16].

It is likely that the phytochemicals that are responsible for the bioactivities of medicinal plant extracts could also be involved in the synthesis of the biogenic metal nanoparticles. A study by Aboyewa et al. (2021) demonstrated that the compound mangiferin, which is a xanthonoid that is found abundantly in extracts of the *Cyclopia intermedia* plant, can be used to produce gold nanoparticles, which have similar physicochemical properties to those produced using the water extract of *C. intermedia* [17]. The nanoparticles produced from such plant extracts may therefore have bioactivities that are similar to those of the extract. *C. orbiculata* extracts contain flavonoids, phenols, saponins, tannins, triterpene steroids and reducing sugars, which contribute to its biological activities [1]. It is therefore plausible to expect that the silver nanoparticles (AgNPs) produced from *C. orbiculata* extracts may have antibacterial and immunomodulatory effects.

Nanomaterials are widely used in different biomedical applications and research. They are incorporated in targeted therapy, drug delivery and in the diagnosis of various diseases [18]. Among metal nanoparticles, which include, but are not limited to, gold, copper, platinum and titanium nanoparticles, AgNPs are known for their antibacterial, antifungal and immunomodulatory activities [13,18]. Antimicrobial resistance is the ability of microbes to resist the effects of antimicrobial drugs [19]. The emergence of antibiotic resistant microorganisms is a global health challenge, rendering common infections difficult to treat. These microorganisms can lead to prolonged illnesses, disability, death and an associated financial burden. Biogenic nanoparticle could be used as alternative antimicrobial agents. Plant derived AgNPs with immunomodulatory and/or antimicrobial properties can contribute to the treatment of resistant infections or chronic inflammation [20].

Inflammation is an essential response triggered by the body’s immune systems to fight infections. However, chronic and uncontrolled inflammation in diseases such as cancer, rheumatoid arthritis, asthma and diabetes is associated with adverse effects. Pro-inflammatory cytokines promote inflammation; however, excessive or uncontrolled production of these cytokines can lead to the development of chronic inflammatory conditions such as chronic obstructive pulmonary disorder, osteoarthritis and cancer [21,22]. Biogenic gold nanoparticles produced from the *H. hemerocallidea* plant have been shown to possess anti-inflammatory properties by reducing the levels of several pro-inflammatory cytokines (TNF-α, IL-6 and IL-1β) [23].

This study investigated the use of *C. orbiculata* aqueous extract to synthesize AgNPs. The reaction parameters of AgNPs synthesis such as time, temperature, silver nitrate (AgNO_3_) and plant extract concentrations were studied in order to optimise the synthesis of the AgNPs from *C. orbiculata* (*Cotyledon*-AgNPs). The physicochemical properties of the *Cotyledon*-AgNPs were characterised. The antimicrobial activity against common skin pathogens (*S. aureus*, *Staphylococcus epidermidis*, Methicillin Resistance *Staphylococcus aureus* (MRSA), *Pseudomonas aeruginosa* and *C. albicans*) and the immunomodulatory effects of *Cotyledon*-AgNPs were determined. Results obtained from this study could potentially lay a foundation for the discovery of new antimicrobial and anti-inflammatory drugs. 

## 2. Materials and Methods

### 2.1. Materials

Fresh *C. orbiculata* plants were purchased from Van Der Berg Garden Village nursery in Stellenbosch, Cape Town, South Africa. AgNO_3_ (99%) was obtained from ACE chemicals (Johannesburg, South Africa). Alamar blue was obtained from ThermoFisher Scientific (Waltham, MA, USA). Lipopolysaccharides (LPS), phorbol 12-myristate 13-acetate (PMA), WST-1 reagent, Roswell Park Memorial Institute (RPMI) medium, Müller-Hinton broth (MHB), Yeast Peptone broth (YPB), Luria-Bertani broth (LB broth), Phosphate-buffered saline (PBS), Fetal bovine serum (FBS), Pen-strep (penicillin and streptomycin), 0.45 µm syringe filters and Whatman filter paper No.1 were obtained from Sigma-Aldrich (St. Louis, Mo, USA). The cytokine ELISA kits were from Bioo Scientific, PerkinElmer (Austin, TX, USA).

### 2.2. Methods

#### 2.2.1. Plant Extract Preparation

The fresh *C. orbiculata* leaves were cut from the whole plant and washed with distilled water. The fresh leaves (300 g) were then cut into small pieces, blended and macerated in 600 mL of distilled water overnight. Thereafter, the extracts were filtered using Whatman filter paper No.1, followed by microfiltration using 0.45 µm filters. The extract was then concentrated by freeze drying and stored at 4 °C until use.

#### 2.2.2. Synthesis of *Cotyledon*-AgNPs

The synthesis of *Cotyledon*-AgNPs was performed according to the methodology reported before, with modifications [16]. In short, 5 mL of AgNO_3_ solution (1 mM and 3 mM) was mixed with 1 mL of the different concentrations of plant extract in glass tubes (1.5, 3, 6, 12, 24 and 48 mg/mL). These mixtures were incubated at 25 °C (room temperature) or 70 °C while stirring for 0.5, 1, 2 or 3 h in the dark. The *Cotyledon*-AgNPs were then purified by centrifugation at 10,000 rpm (Centrifuge 5417R, rotor (F45-30-11), Eppendorf AG, Hamburg, Germany) for 10 min, and then the pellet was re-suspended in distilled water. This process was repeated three times.

#### 2.2.3. Stability Testing of *Cotyledon*-AgNPs

The stability of the *Cotyledon*-AgNPs was evaluated in different biological media and buffers used in this study (RPMI, MHB, YPB, PBS and LB broth). This assay was performed immediately after the *Cotyledon*-AgNPs were purified. In glass test tubes, 250 µL of aqueous solutions of *Cotyledon*-AgNPs were mixed with the same volume of the medium or buffer. The tubes were incubated at 25 °C or 37 °C for 24 h. The stability of these nanoparticles was evaluated by measuring changes in their Ultraviolet-Visible spectroscopy (UV-Vis) spectra.

#### 2.2.4. Characterisation of *Cotyledon*-AgNPs

##### UV-Vis

The formation of *Cotyledon*-AgNPs was confirmed by observing their Surface Plasmon Resonance (SPR) peaks using UV-Visible spectroscopy (POLARstar Omega microplate reader (BMG-Labtech, Ortenberg, Germany)) at a wavelength range of 250 to 800 nm.

##### Dynamic Light Scattering (DLS)

The DLS analysis was performed using Zetasizer Nano ZS90 (Malvern, UK) to determine particle size distribution, zeta potential and Polydispersity Index (PDI) values of the *Cotyledon*-AgNPs.

##### High Resolution Transmission Electron Microscopy (HR-TEM)

The morphology and the particle size distribution of *Cotyledon*-AgNPs were determined by HR-TEM analysis using a FEI Tecnai G2 F20 field-emission gun (Field Electron and Ion Company, Hillsboro, OR, USA). For the preparation for HR-TEM, a drop of *Cotyledon*-AgNPs solution was loaded onto a carbon coated copper grid. The grid was dried under a Xenon lamp for 10 min before analysis. The particle size distribution was determined using Image J software. Selected Area Electron Diffraction (SAED) and Energy-dispersive X-ray spectroscopy (EDX) analyses were also analysed on the same samples using HR-TEM.

#### 2.2.5. Antimicrobial Inhibition

##### MIC

The antimicrobial properties of the *Cotyledon*-AgNPs were evaluated by determining their MIC using a microdilution assay [24]. The MIC was determined against *S. aureus* (ATCC 25923), MRSA (33591), *S. epidermidis* (ATCC 12228), *P. aeruginosa* (ATCC 27853) and *C. albicans* (ATCC 10231). Briefly, 50 μL of *Cotyledon*-AgNPs (in LB broth) were added in 96-well plates with decreasing concentrations (320, 160, 80, 40, 20, 10, 5 and 2.5 µg/mL).

Microbial suspensions of the bacteria and the fungus were cultured in LB broth and diluted until they reached a 0.5 McFarland standard (standard is approximately 1 × 10^8^ CFU/mL) [24]. A volume of 50 μL of the microbial suspensions was added to each well containing LB broth and AgNPs. The plates were sealed and incubated for 24 h at 37 °C. Ampicillin (2.5 mg/mL) and Fluconazole (10 mg/mL) were used as positive controls for the bacteria and the fungus, respectively, while sterile deionized water was used as the negative control.

After incubation, 10 µL of Alamar Blue was added to each well and the plate was incubated for 3 h in the dark. In the presence of viable bacteria, the non-fluorescent Alamar Blue dye (resazurin) was reduced to resofurin, a pink compound that was highly fluorescent [25]. Therefore, the intensity of the pink colour and the fluorescence was proportional to the number of viable cells present. The MIC was then determined using a spectrophotometer at a fluorescence excitation/emission wavelength of 530–560/590 nm. The screening was performed in triplicates.

##### Minimum Bactericidal Concentration (MBC)/Minimum Fungicidal Concentration (MFC)

This assay was conducted according to the previously reported methodology, with modifications [26]. The MBC and MFC were determined by sub-culturing a loopful of media from the wells that showed no growth of microorganisms during MIC determination. The culture plates were incubated at 37 °C for 24 h. The MBC/MFC was recorded as the lowest concentration at which no growth was observed on the culture plates.

#### 2.2.6. Immunomodulatory Studies

##### Cell Culture

The human monocytic leukaemia cell line, THP-1, was obtained from ATCC (TIB-202), (Manassas, VA, USA). THP-1 cells were cultured in RPMI (1640) medium containing 10 % FBS and 1 % Pen-strep. The cells were incubated at 37 °C in a humidified atmosphere of 5 % CO_2_ in a SL SHEL LAB incubator (Sheldon manufacturing, Cornelius, OR, USA).

##### Differentiation of THP-1 Cells

THP-1 monocytes were differentiated into macrophages using PMA. The THP-1 monocytes were seeded in 24-well plates (500 µL) at a density of 2 × 10^5^ cells/mL and were treated with 100 nM PMA. These cells were incubated at 37 °C in a humidified atmosphere of 5 % CO_2_. After 3 days of incubation, the culture media was replaced with PMA-free media (resting phase) for another 24 h [27].

##### Cell Viability

Cell viability was evaluated using the WST-1 assay, according to the manufacturer’s protocol. This assay was conducted to determine the toxicity of the *Cotyledon*-AgNPs on the differentiated THP-1 macrophages. Using 96-well cell culture plates, the macrophages were treated with different concentrations of the *Cotyledon*-AgNPs (20, 10, 5, 2.5, 1.3, 0.6 and 0.3 µg/mL). The cells were incubated for 24 h at 37 °C in a humidified atmosphere of 5 % CO_2_. After incubation, the medium was removed from the wells and replaced with a medium containing 10 % WST-1 reagent. The cells were incubated for another 3 h, and the absorbance of the culture media was measured at 440 nm (reference 630 nm) using the POLARstar Omega plate reader (BMG-Labtech, Ortenberg, Germany). Cell viability was expressed as a percentage of the absorbance of treated cells to control (untreated) cells.

##### Determination of Cytokine Responses

Stimulation of macrophages for cytokine determination was performed using LPS from *Escherichia coli* 0111:B4. PMA-differentiated THP-1 macrophages were stimulated with 1 µg/mL LPS [28] for 6 h in 24-well plates. After removal of LPS from cell culture, the stimulated cells were incubated with the *Cotyledon*-AgNPs at 37 °C for 24 h. The LPS control was treated with LPS only. After 24 h incubation, the cell supernatants were transferred into Eppendorf tubes and centrifuged at 1500 rpm for 10 min. The supernatants were then collected and stored at −80 °C until measurement of cytokines. The production levels of the cytokines, IL-1β, IL-6 and TNF-α were determined using enzyme-linked immunosorbent assay (ELISA) kits, according to the manufacturers’ instructions.

##### Statistical Analysis

Statistical analysis of the data was conducted using GraphPad Prism 6 software. The results were expressed as mean ± standard error of the mean (SEM). The significance of the cell viability and immunomodulatory effects of *Cotyledon*-AgNPs were determined using the two-way analysis of variance (ANOVA). Values were considered to be statistically significant at *p* < 0.0001.

## 3. Results and Discussion

### 3.1. Synthesis of Cotyledon-AgNPs

Generally, AgNPs are formed by the reduction of silver salt to silver ions (Ag^+^ to Ag^0^) [13]. The phytochemicals in the plant extracts, such as alkaloids, terpenoids and flavonoids, act as reducing agents in the synthesis of AgNPs [29]. Plants differ in their phytochemical composition and, therefore, their ability to synthesize nanoparticles also differs. It is therefore imperative to optimise the synthesis of nanoparticles from each plant extract. The synthesis of *Cotyledon*-AgNPs was optimised by varying different reaction parameters such as temperature, time and the concentration of both AgNO_3_ and the plant extract solutions.

**Figure 1:**
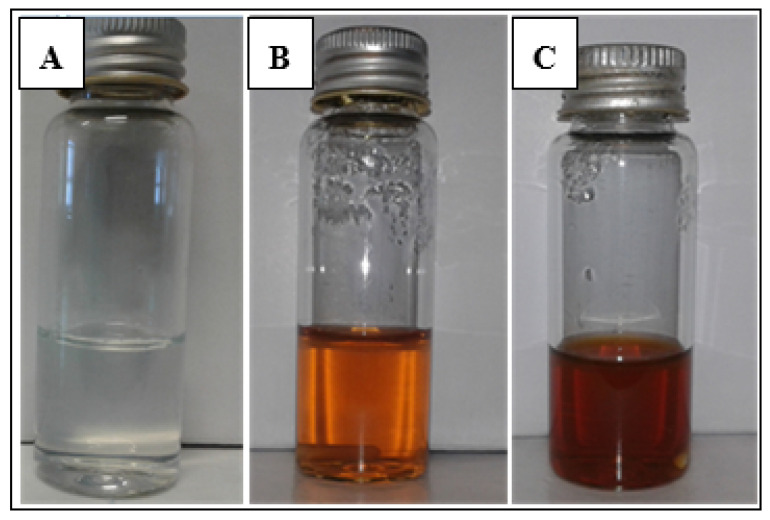
Colour change of *Cotyledon*-AgNPs at (**A**) 0 min (**B**) 30 min and (**C**) 1 h.

The synthesis of AgNPs was indicated by a colour change in the reaction mixture (AgNO_3_ and *C. orbiculata* aqueous extract solution). Immediately after the addition of the plant extract to the AgNO_3_ solution, the mixture was colourless (Figure 1A). However, after 30 min, the colour changed to orange, which intensified to a brown colour after 1 h from the start of the reaction, as shown in Figure 1B and Figure 1C, respectively. The change in colour of the solution is due to the formation of AgNPs [30]. AgNPs are known to exhibit a yellowish-brown colour due to the excitation of their SPR vibrations [31].

The synthesis of AgNPs was further confirmed by measuring the UV-Vis spectra between 250–800 nm. The maximum absorbance (λ_max_) of the *Cotyledon*-AgNPs was around 420 nm, which is characteristic of AgNPs. AgNPs exhibit an SPR at around 400–500 nm [32]. SPR of lower wavelengths suggest that smaller nanoparticles were formed and vice versa [33].

### 3.2. The Effect of AgNO_3_ Concentration on the Synthesis of Cotyledon-AgNPs

**Figure 2:**
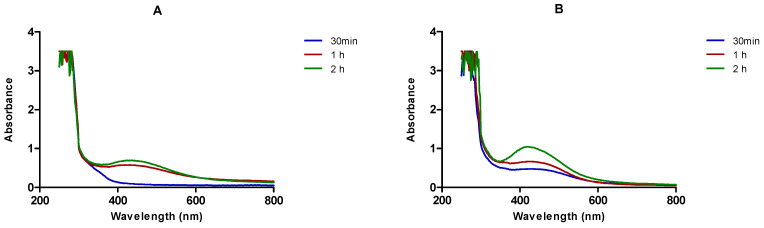
A comparison of the UV-Vis absorption spectra of *Cotyledon*-AgNPs, synthesized using (**A**) 1 mM and (**B**) 3 mM AgNO_3_ solution.

The synthesis of *Cotyledon*-AgNPs was optimized using two AgNO_3_ concentrations (1 mM and 3 mM). These two concentrations are commonly reported in the literature for the synthesis of AgNPs from plants [4,14,34,35,36]. Figure 2 shows the effect of AgNO_3_ concentration on the synthesis of *Cotyledon*-AgNPs. The use of 3 mM AgNO_3_ produced AgNPs with an intense brown colour and sharper absorbance peaks. Moreover, 3 mM AgNO_3_ produced a larger amount of AgNPs. This was evident from the height of the absorbance peaks, which gives an indication of the number of the nanoparticles produced. It is known that absorbance is directly proportional to the concentration of AgNPs in a solution [16]. Subsequent nanoparticle synthesis was therefore performed using 3 mM AgNO_3._

### 3.3. The Effect of C. orbiculata Aqueous Extract Concentration on the Synthesis of Cotyledon-AgNPs

Another reaction parameter evaluated was the concentration of the plant extract. Of the different plant extract concentrations tested (48, 24, 12, 6, 3, and 1.5 mg/mL) only three concentrations (6, 3, and 1.5 mg/mL) gave nanoparticles, as determined by the change in the colour of the respective reaction mixtures after the addition of AgNO_3_ (3 mM). This further suggests that extremely high concentrations of the plant extract hinder the formation of nanoparticles.

**Figure 3:**
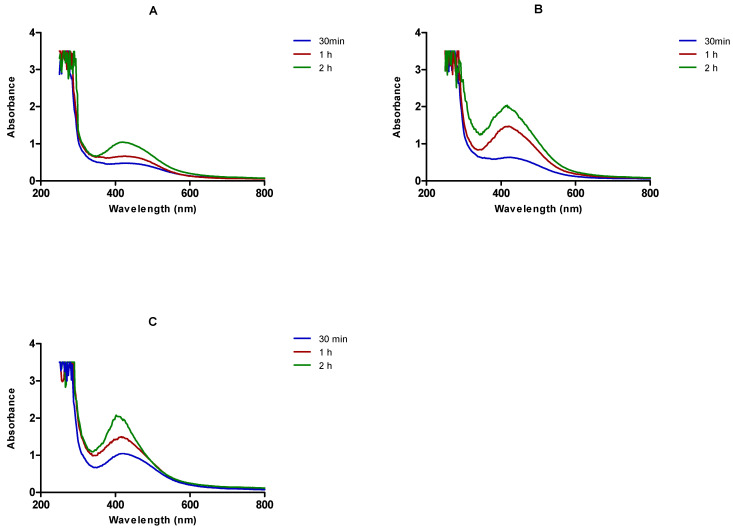
Changes in the UV- Vis absorption spectra of *Cotyledon*-AgNPs over time, using (**A**) 1.5, (**B**) 3, and (**C**) 6 mg/mL of *C. orbiculata* plant extract.

It can be observed in Figure 3 that the absorption spectra of the AgNPs became sharper with the increase in plant extract concentration (from 1.5 to 6 mg/mL). No absorbance was recorded when using 48, 24 and 12 mg/mL of the plant aqueous extract (data not shown). The sharpness of these peaks and the blue-shift of the λ_max_ suggest the formation of smaller and monodispersed nanoparticles by increasing the concentrations of the plant extract [37]. A study by Benakashani and colleagues (2016) investigated the effects of varying the concentrations of plant extract on AgNPs synthesis. Their results indicated that the increase in the plant extract concentration led to the formation of nanoparticles with a sharper absorption peak [12]. This is in agreement with the findings in this study, where an increase in *C. orbiculata* extract concentration (from 1.5 to 6 mg/mL) enhanced the formation of AgNPs.

Increasing the plant extract concentrations also changed the intensity of the reaction mixture’s colour, and produced AgNPs at a faster rate than the lower concentrations of the plant extract. This is as also indicated by the increase in the absorbance of the UV-Vis spectra. As shown in Figure 3, 3 mg/mL of *C. orbiculata* extract produced AgNPs with an absorbance reading of 1.47 after 1 h from the start of the reaction, whereas 1.5 mg/mL of the extract produced AgNPs with a lower absorbance reading of 0.66 within the same time frame. This might be because of the availability of higher amounts of phytochemicals when using high concentrations of the plant extract, which, therefore, increased the reduction power in the reaction medium, leading to a reduction of more AgNO_3_ molecules in a short time [15,38,39]. This can be observed by measuring the UV-Vis spectra of the AgNPs over different time periods from the start of the reaction (Figure 3).

### 3.4. The Effect of Temperature on the Synthesis of Cotyledon-AgNPs

Temperature is another important factor in the synthesis of nanoparticles. High temperature is suggested to facilitate the synthesis of nanostructures [40]. It has also been found that synthesis temperature can affect the size, shape and the yield of nanoparticles synthesized using plant extracts [41,42]. A study on nanoparticle synthesis using *C. sinensis* (sweet orange) peel extract showed a decrease in the size of AgNPs with increasing temperature [43]. At 25 °C, *C. sinensis* aqueous peel extract produced particles with a size of 35 nm but, at 60 °C, particle size decreased to 10 nm. In this study, however, no nanoparticles were produced at 25 °C. The synthesis was only accomplished at 70 °C. This is also in agreement with a study by Elbagory and colleagues (2016), in which aqueous extracts of *Asparagus rubicundus*, *Aspalathus hispida* and *Dicerothamnus rhinocertis* produced gold nanoparticles at 70 °C only [16]. This might suggest that certain reducing phytochemicals in plants require higher temperatures to initiate the reduction process [41].

### 3.5. The Effect of Reaction Time on the Synthesis of Cotyledon-AgNPs

Reaction time can also affect the synthesis of AgNPs. A colour change was observed within 30 min of *Cotyledon*-AgNPs synthesis (Figure 1). The change in colour intensity of the reaction mixture with time is an indication of the increased amount of AgNPs formed [44]. Indeed, in Figure 3, the height of the absorbance peaks increased with time, indicating an increase in the concentration of AgNPs. At 30 min, the absorbance reading was 0.63, which increased to 1.47 and 2 after 1 and 2 h, respectively (Figure 3B).

### 3.6. DLS Analysis

A DLS analysis was used to measure size distribution, zeta potential and PDI values of the *Cotyledon*-AgNPs (Table 1). The PDI is a measure of the width of molecular weight distribution and has a value between 0 and 1. Nanoparticles with a PDI value of less than 0.2 are considered monodispersed [45,46]. The increase in the PDI value indicates an increase in the broadness of the particle size distribution [47]. The *Cotyledon*-AgNPs have PDI values of 0.07, 0.15 and 0.1 for AgNPs synthesized using 6, 3 and 1.5 mg/mL of *C. orbiculata* plant extract. Hence, these nanoparticles are monodispersed.

The average particle sizes were 137 ± 2, 110 ± 2, and 106 ± 2 nm for AgNPs synthesized using 1.5, 3 and 6 mg/mL of *C. orbiculata* extract, respectively. The results show that the average size of the *Cotyledon*-AgNPs decreased as the concentration of the *C. orbiculata* extract increased. Increasing the extract concentration increases the reducing agents in the sample, thus promoting faster AgNO_3_ reduction and formation of nanoparticles which tend to be smaller in size [48]. This corresponds to the findings in Figure 3, where the spectrum of the nanoparticles became sharper with the increase of the plant extract concentration. Sharp absorption spectrum of nanoparticles suggests smaller nanoparticles [49]. The data agree with previous studies conducted using banana peel extract [15] and *C. spinosa* extract [12]. However, in a study performed by Verma and Mehata (2016), the size of the AgNPs synthesized using neem leaf broth increased with increases in extract concentration [39].

Zeta potential measures the charge of the AgNPs, which can be used to determine their stability. The charge reflects the repulsive forces between the nanoparticles [50]. The *Cotyledon*-AgNPs had an average zeta potential of −19 mV (Table 1). This indicates that the *Cotyledon*-AgNPs are relatively stable and dispersed due to repulsion forces [51]. Metal nanoparticles with large negative zeta potential repel each other, and therefore do not aggregate. However, those with low zeta potential values can easily aggregate due to the absence of strong repulsive forces [52]. According to Ardani et al. (2017), nanoparticles with positive or negative zeta potential values between ±10 to 20 mV are considered relatively stable [53]. Based on this, it can be concluded that the nanoparticles shown in Table 1 are all relatively stable.

### 3.7. Stability of the Cotyledon-AgNPs in Biological Media

Nanoparticles should remain stable in different biological environments for any potential biomedical application. Nanoparticles with low stability can easily aggregate, which, consequently, can alter their intended properties [54]. Aggregation of nanomaterials may affect their toxicity to different cells, which may adversely affect their suitability for applications in therapeutics [55,56]. Therefore, the stability of *Cotyledon*-AgNPs was evaluated in several biological media.

**Figure 4:**
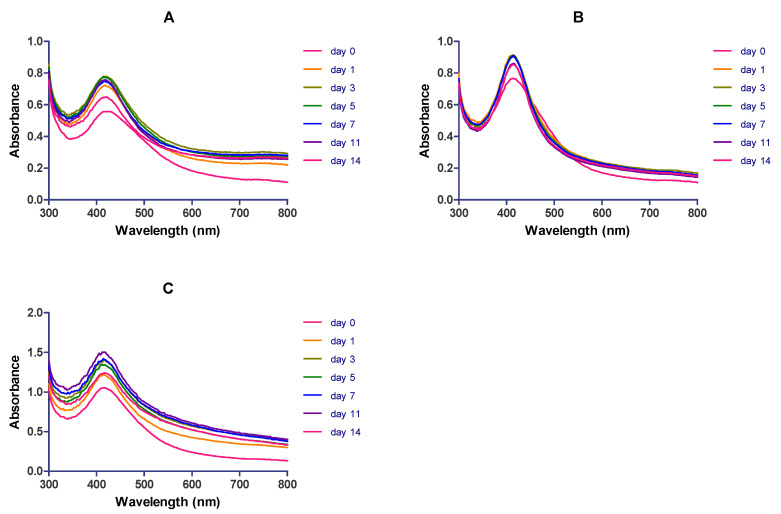
The UV-Vis spectra record of *Cotyledon*-AgNPs stored at 25 °C in the dark over a 14-day period. The *Cotyledon*-AgNPs were synthesized using (**A**) 1.5, (**B**) 3 and (**C**) 6 mg/mL of *C. orbiculata* plant extract.

The aqueous solution of *Cotyledon*-AgNPs was kept at 25 °C in the dark and their stability was evaluated over a period of 14 days. Figure 4 shows the effects of storage time on the UV-Vis spectra of *Cotyledon*-AgNPs, synthesized using different plant extract concentrations. *Cotyledon*-AgNPs synthesized using 3 mg/mL plant extract were more stable compared to those synthesized using 1.5 and 6 mg/mL. This can be seen in their absorption spectra, which did not change during the 14-day period, indicating their stability [57]. The *Cotyledon*-AgNPs in this study were therefore synthesized using 3 mg/mL of plant extract and 3 mM AgNO_3_ at 70 °C for 2 h.

**Figure 5:**
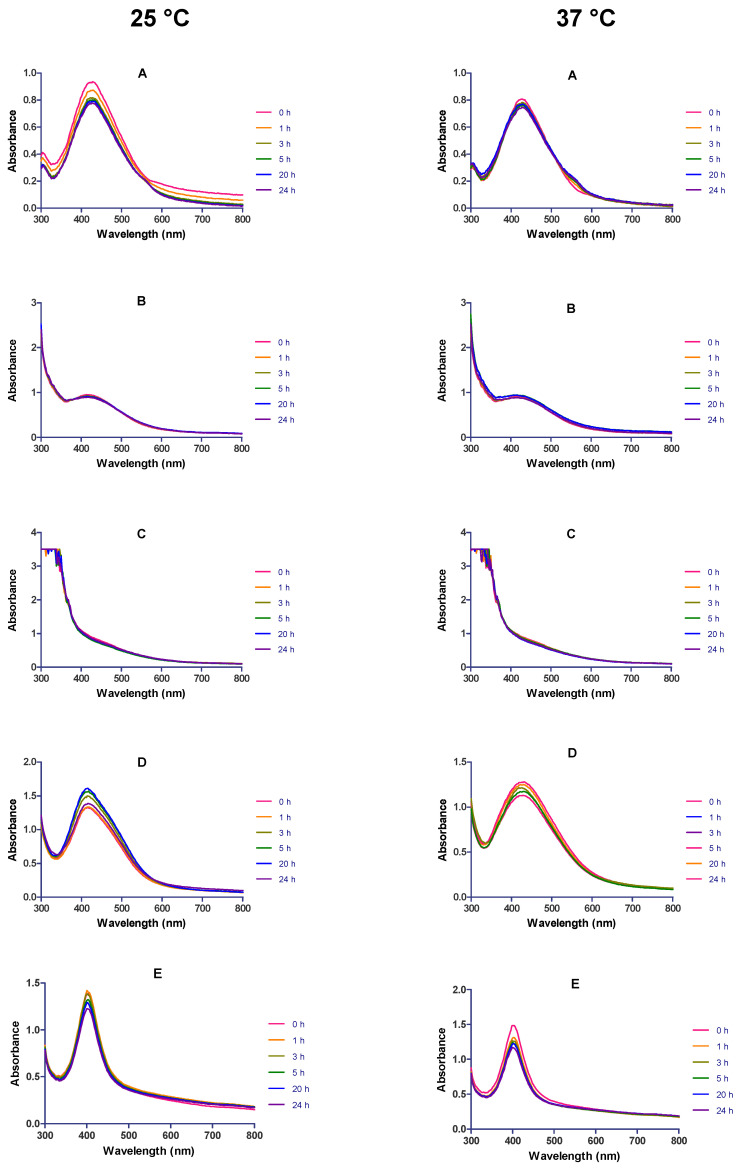
The UV-Vis spectra of *Cotyledon*-AgNPs, recorded over a 24 h period (at 25 and 37 °C) after mixing with different biological media; (**A**) RPMI, (**B**) MHB, (**C**) YPB, (**D**) LB broth and (**E**) PBS.

The stability of *Cotyledon*-AgNPs was also evaluated in RPMI, MHB, YPB, LB broth and PBS over a 24 h period. These media and buffers were selected because they are used in several biological assays. The addition of *Cotyledon*-AgNPs to RPMI, LB broth and PBS did not change their UV-Vis absorption spectra (Figure 5A,D,E). The stability of the *Cotyledon*-AgNPs was not only evaluated at room temperature (25 °C), but also at 37 °C to observe the effect of biological assay conditions on their stability. Incubation of most biological assays, such as cell culture, cell viability assays and bacterial culture, is conducted under these temperatures. The UV-Vis absorption spectra showed that the SPR characteristics of the nanoparticles did not change throughout the incubation time in RPMI, LB broth and PBS, implying that they are stable in these solutions. On the other hand, the *Cotyledon*-AgNPs were not stable in MHB and YPB at both temperatures (Figure 5B,C). The addition of the *Cotyledon*-AgNPs to YPB and MHB resulted in the flattening of the absorption spectra, probably as a result of nanoparticle aggregation. MHB and YPD have a high protein content and a lower concentration of inorganic salts compared to LB broth [58]. It is reported that high protein contents in bacterial growth media can negatively affect the colloidal stability of NPs, leading to their agglomeration [59]. The use of YPB and MHB was therefore avoided in the biological evaluation assays.

### 3.8. HR-TEM, SAED and EDX

**Figure 6:**
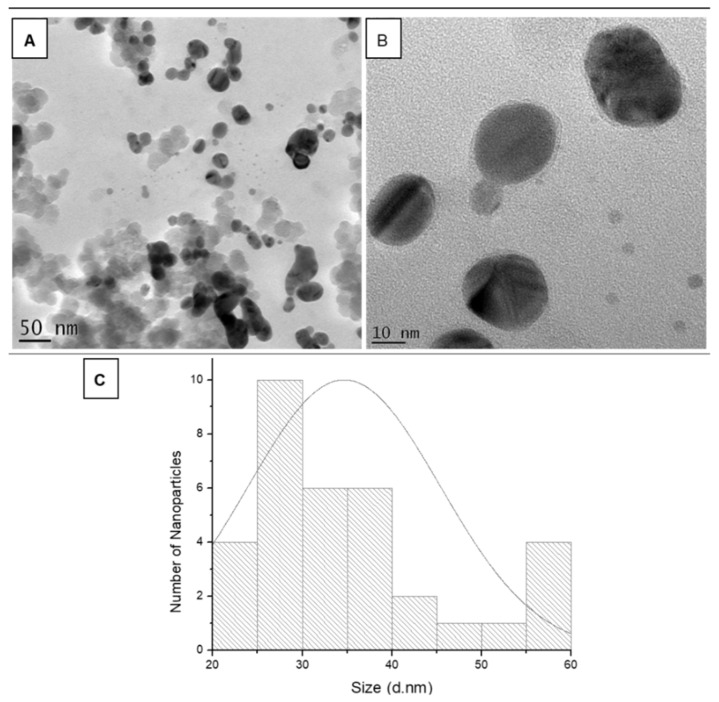
HR-TEM analysis of *Cotyledon*-AgNPs. HR-TEM images of *Cotyledon*-AgNPs at a (**A**) 50 and (**B**) 10 nm scale. (**C**) represents the size distribution for the *Cotyledon*-AgNPs obtained from the HR-TEM images.

The morphology and size of the *Cotyledon*-AgNPs were analysed by HR-TEM. The HR-TEM images showed that the *Cotyledon*-AgNPs were mostly spherical (Figure 6). The size of the *Cotyledon*-AgNPs ranged from 20 to 40 nm, with a few larger particles of approximately 60 nm (Figure 6C). However, the average nanoparticle sizes recorded for DLS in Table 1 are much larger than the nanoparticle sizes shown by HR-TEM. This is because DLS measures the hydrodynamic sizes of nanoparticles, which includes the surrounding organic layer, whereas HR-TEM only measures the size of the inorganic core [52,60,61]. Similar results were also obtained in a study by Kittler et al. (2010), in which the DLS recorded higher average size for PVP-coated AgNPs compared to average size obtained from electron microscopy [62].

**Figure 7:**
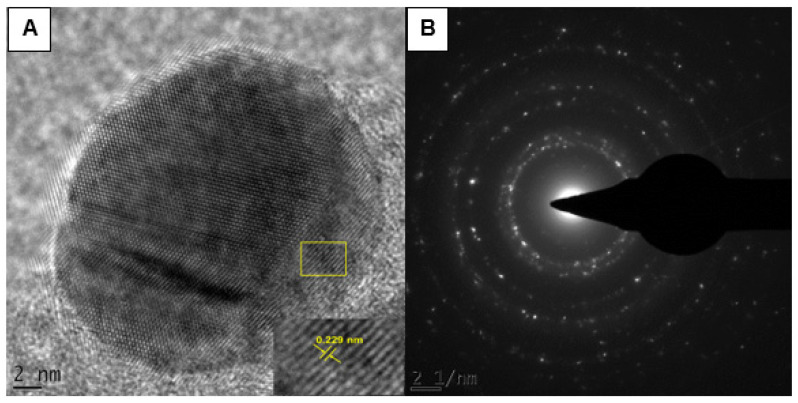
The HR-TEM images of the *Cotyledon*-AgNPs showing (**A**) the lattice fringes and (**B**) the SAED pattern.

**Figure 8:**
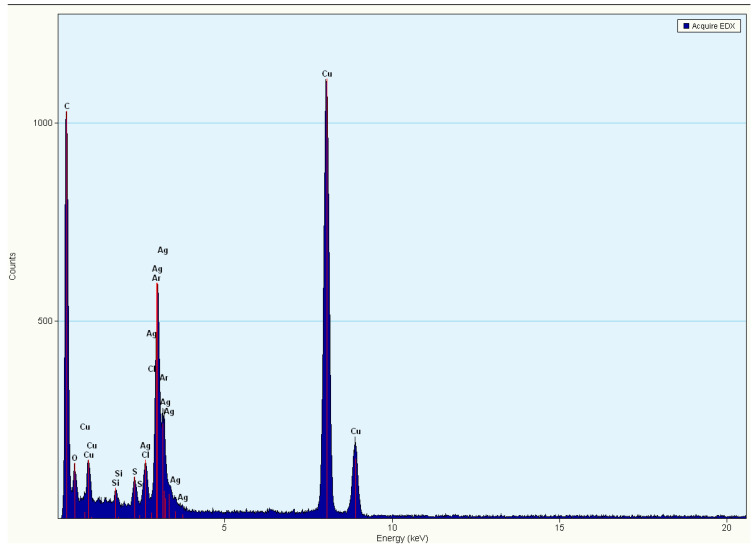
EDX spectrum of the *Cotyledon*-AgNPs.

The lattice fringes on the particles indicate the crystalline nature of the *Cotyledon*-AgNPs (Figure 7A). The fringe spacing was found to be 0.229 nm. Crystallinity of the AgNPs was also confirmed by the SAED pattern shown in Figure 7B. The rings were indexed and were found to correspond to the (111), (200), (220) and (311) crystalline planes of the face-centred cubic of metallic silver [63]. The EDX analysis, which gives the elemental composition of nanoparticles, confirmed that *Cotyledon*-AgNPs contained silver (Figure 8). The optical adsorption peak observed at 3 keV is due to the presence of silver [64,65,66]. Adsorption peaks were also observed for carbon, oxygen, silicon, sulphur, chloride and copper. The presence of carbon, oxygen, chloride and sulphur is likely to be from the phytochemicals involved in the reduction and the capping processes during nanoparticle synthesis, as proposed before [16]. The presence of copper and silicon can be ascribed to the HR-TEM grid onto which the sample was placed [67].

The morphology and crystallinity of the *Cotyledon*-AgNPs produced in this study were similar to those AgNPs reported in several other studies using extracts of *Crocus sativus* L. [68], *D. stramonium* [13], *Urtica dioica* [69], *Azadirachta indica* [39], Olive oil [70] and tea leaves [71].

### 3.9. Antimicrobial Activity of Cotyledon-AgNPs

The antimicrobial activity of *Cotyledon*-AgNPs was investigated against skin pathogens, namely *S. aureus*, *S. epidermidis*, MRSA, *P. aeruginosa* and *C. albicans.* This was conducted by determining the MIC and MBC/MFC of AgNPs against the aforementioned microorganisms. *Cotyledon*-AgNPs exhibited antibacterial and antifungal activity against all the organisms tested in this study, as indicated in Table 2. The aqueous extract of *C. orbiculata* did not display any significant antimicrobial activity at the tested concentrations. However, *Cotyledon*-AgNPs were mostly active against *P. aeruginosa*, with MIC and MBC values of 5 and 20 µg/mL, respectively. The lowest activity recorded for *Cotyledon*-AgNPs was against *C. albicans*, with MIC and MFC values of 80 and 160 µg/mL, respectively. *Cotyledon*-AgNPs exhibited the same inhibitory activity against *S. aureus* and *S. epidermidis*, with an MIC value of 20 µg/mL. However, their bactericidal effects differed, as shown by the different MBC values against these microorganisms (MBC against *S. aureus* and *S. epidermidis* was 40 and 20 µg/mL, respectively). The antimicrobial activity of *Cotyledon*-AgNPs compared extremely well with the positive controls (ampicillin and fluconazole). In fact, the antimicrobial activity of *Cotyledon*-AgNPs against MRSA and *P. aeruginosa* was higher than both controls used. While the MIC for ampicillin was 310 µg/mL for MRSA and >1000 µg/mL for *P. aeruginosa*, the MICs for *Cotyledon*-AgNPs were 40 and 5 µg/mL against MRSA and *P. aeruginosa*, respectively.

The *Cotyledon*-AgNPs were more active against the Gram-negative bacteria (*P. aeruginosa*) as compared to the Gram-positive bacteria (*S. epidermidis*, *S. aureus* and MRSA). This is likely due to the different membrane structures and compositions in the cell wall of the microorganisms [13]. Gram-positive bacteria have a thick layer of peptidoglycan in their cell wall, whereas, in Gram-negative bacteria, the cell wall contains a thin peptidoglycan layer [72]. These results are in agreement with other findings in literature where Gram-negative bacteria were more susceptible to AgNPs than Gram-positive bacteria [12,13,15]. In this study, the *Cotyledon*-AgNPs also exhibited better antibacterial activity than the antifungal activity, which corresponds to the findings reported previously [15,73].

The high antibacterial activity of AgNPs is attributable to their large surface area, which provides better contact of the nanoparticles with the cell wall of microorganisms [44]. Other mechanisms of AgNPs antibacterial activity include their ability to bind to phosphorus and sulfur-containing cell constituents such as DNA and some proteins, leading to their degradation. AgNPs also release silver ions into the bacteria cell wall, which can cause death of the bacteria [74].

### 3.10. Cell Culture and Differentiation of THP-1

The human monocytes THP-1 cells, used herein to evaluate immunomodulatory activity, were cultured in RPMI and differentiated into macrophages using 100 nM of PMA. PMA, a cognate of diacylglycerol, is an activator of protein kinase C that is used in cell differentiation [75,76]. Upon differentiation, there are morphological changes that occur within the cells. In comparison to undifferentiated THP-1, differentiated THP-1 cells are larger, adherent and show expansion of the cytoplasm and of cytoplasmic organelles such as mitochondria and lysosomes [77]. Other differentiation stimuli, such as 1.25-dihydroxyvitamin D3, have been reported. However, the differentiation of monocytes with PMA was shown to have more resemblance to the phenotype of human tissue macrophages [78].

### 3.11. Cell Viability of THP-1 Cells Treated with Cotyledon-AgNPs

Cell viability assays are crucial for the determination of the cytotoxic effects of compounds on different cell lines in vitro. In this study, a WST-1 assay was used to determine the cytotoxicity of *Cotyledon*-AgNPs on the differentiated THP-1 macrophages. This assay is based on the conversion of a water-soluble tetrazolium salt into formazan by the mitochondrial dehydrogenases of the viable cells. Therefore, the number of viable cells can be quantified by detecting the amount of formazan produced [79].

**Figure 9:**
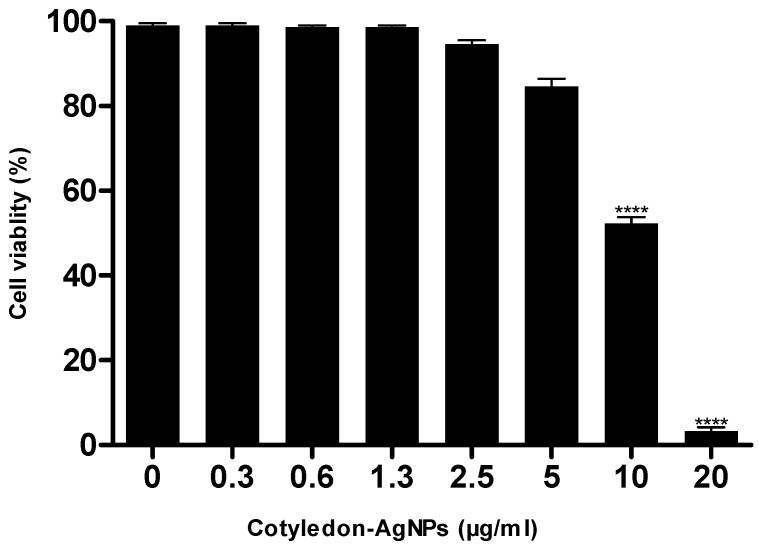
Effects of *Cotyledon*-AgNPs on the viability of differentiated THP-1 macrophages. Each value represents mean ± SEM; statistical significance of the *Cotyledon*-AgNPs-treated cells when compared to the untreated control cells is indicated with **** for *p* < 0.0001.

The *Cotyledon*-AgNPs showed no toxicity to THP-1 cells at concentrations below 10 µg/mL (Figure 9). The percentage viability of the differentiated THP-1 macrophages was 84 %, 52 % and 2 % when the cells were treated with 5, 10 and 20 µg/mL of *Cotyledon*-AgNPs, respectively. Thus, 5 µg/mL of *Cotyledon*-AgNPs was the least toxic concentration and was used to evaluate the immunomodulatory effects of the nanoparticles.

### 3.12. Cytokine Secretion

**Figure 10:**
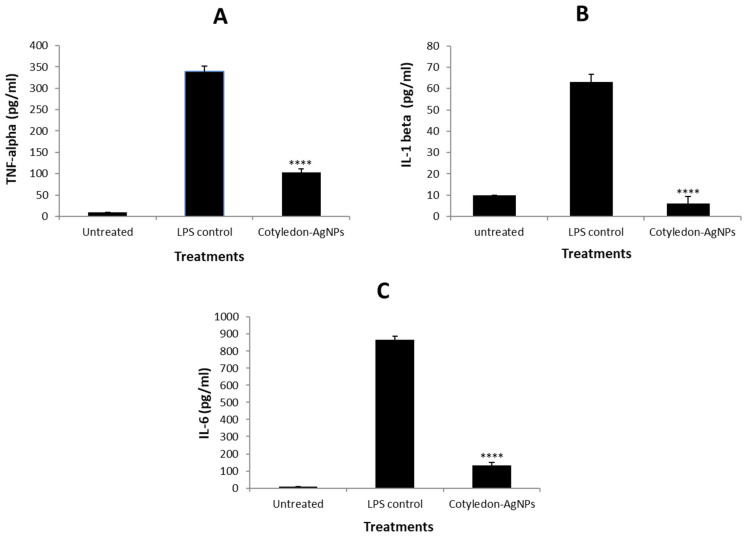
Effects of *Cotyledon*-AgNPs on cytokine secretion in LPS stimulated THP-1 macrophages, differentiated using PMA. (**A**) represents TNF-α, (**B**) represents IL-1β, and (**C**) represents IL-6. Each value represents mean ± SEM; statistical significance of the *Cotyledon*-AgNPs-treated cells to the LPS controls is indicated with **** for *p* < 0.0001.

ELISA was used to determine the effects of *Cotyledon*-AgNPs on cytokine production in the differentiated THP-1 cells. After differentiation with PMA, the macrophages were stimulated with LPS before exposure to 5 µg/mL *Cotyledon*-AgNPs. LPS are biologically active substances found in the outer membrane of Gram-negative bacteria [80]. Exposure of THP-1 macrophages to LPS stimulates the production of pro-inflammatory cytokines, which are essential for antibacterial defence when produced in appropriate amounts [81]. The levels of cytokine production by the macrophages in the presence of *Cotyledon*-AgNPs are shown in Figure 10. LPS treatment of the THP-1 macrophages resulted in the production of pro-inflammatory cytokines (TNF-α, IL-6 and IL-1β). However, the addition of *Cotyledon*-AgNPs (5 µg/mL) to the macrophages reduced the production levels of these cytokines. The levels of cytokine production in the cells treated with *Cotyledon*-AgNPs was significantly decreased by approximately 3.5-, 7- and 10.5-fold for TNF-α, IL-1β and IL-6, respectively, when compared to the LPS treated cells (Figure 10). Therefore, the results obtained in this study indicate that *Cotyledon*-AgNPs have anti-inflammatory properties, as they decreased the production levels of pro-inflammatory cytokines in LPS stimulated THP-1 macrophages. AgNPs synthesized using Curcumin and *Asparagus racemosus* root extract have shown similar results; they also reduced the levels of proinflammatory cytokines (IL-6 and TNF-α) in THP-1 cells, suggesting that they have some anti-inflammatory activity [82,83]. 

## 4. Conclusions

AgNPs were successfully synthesized using *C. orbiculata* aqueous extract. The synthesis was optimized by varying reaction parameters such as time, temperature, AgNO_3_ and plant extract concentrations. The formulated *Cotyledon*-AgNPs were fully characterized and were found to be stable in different biological media. The antimicrobial study showed that *Cotyledon*-AgNPs exhibited antimicrobial activity against several skin-related pathogens more than *C. orbiculata* aqueous extract. *Cotyledon*-AgNPs also exhibited anti-inflammatory effects by suppressing the production of pro-inflammatory cytokines (IL-1β, IL-6 and TNF-α) in macrophages.

Therefore, this study demonstrates the potential of *Cotyledon*-AgNPs as antimicrobial agents in the treatment of skin infections. The study also shows the potential of *Cotyledon*-AgNPs as agents to control inflammatory disorders that cause chronic inflammation.

## Figures and Tables

**Table 1:** Average size, PDI and zeta potential of the *Cotyledon*-AgNPs synthesized using different concentrations of *C. orbiculata* extract (6, 3 and 1.5 mg/mL) at 70 °C for 2 h.

Parameter	Plant Extract Concentration		
	6 mg/mL	3 mg/mL	1.5 mg/mL
Average size (nm)	106 ± 2	110 ± 2	137 ± 2
PDI	0.07 ± 0.02	0.15 ± 0.33	0.1 ± 0.01
Zeta potential	−19 ± 1.0	−20 ± 1.0	−18 ± 1.0

**Table 2:** The MIC and MBC/MFC (µg/mL) values * of *Cotyledon*-AgNPs and *C. orbiculata* aqueous extract against common skin pathogens.

Microorganisms	*Cotyledon*-AgNPs		*C. orbiculata* Aqueous Extract		Ampicillin/ Fluconazole	
	MIC	MBC/MFC	MIC	MBC/MFC	MIC	MBC/MFC
*S. aureus*	20	40	˃1000	˃1000	20	40
*S. epidermidis*	20	20	˃1000	˃1000	40	40
MRSA	40	80	˃1000	˃1000	310	630
*P. aeruginosa*	5	20	˃1000	˃1000	>1000	˃1000
*C. albicans*	80	160	>1000	˃1000	60	1000

* The values were observed from three experiments which gave identical results.

## Data Availability

Supporting data presented in this study are available on request from the corresponding author.
